# NMR Spectroscopic and Bioinformatic Analyses of the LTBP1 C-Terminus Reveal a Highly Dynamic Domain Organisation

**DOI:** 10.1371/journal.pone.0087125

**Published:** 2014-01-29

**Authors:** Ian B. Robertson, Penny A. Handford, Christina Redfield

**Affiliations:** Department of Biochemistry, University of Oxford, Oxford, United Kingdom; MRC National Institute for Medical Research, United Kingdom

## Abstract

Proteins from the LTBP/fibrillin family perform key structural and functional roles in connective tissues. LTBP1 forms the large latent complex with TGFβ and its propeptide LAP, and sequesters the latent growth factor to the extracellular matrix. Bioinformatics studies suggest the main structural features of the LTBP1 C-terminus are conserved through evolution. NMR studies were carried out on three overlapping C-terminal fragments of LTBP1, comprising four domains with characterised homologues, cbEGF14, TB3, EGF3 and cbEGF15, and three regions with no homology to known structures. The NMR data reveal that the four domains adopt canonical folds, but largely lack the interdomain interactions observed with homologous fibrillin domains; the exception is the EGF3-cbEGF15 domain pair which has a well-defined interdomain interface. ^15^N relaxation studies further demonstrate that the three interdomain regions act as flexible linkers, allowing a wide range of motion between the well-structured domains. This work is consistent with the LTBP1 C-terminus adopting a flexible “knotted rope” structure, which may facilitate cell matrix interactions, and the accessibility to proteases or other factors that could contribute to TGFβ activation.

## Introduction

**Figure 1:**
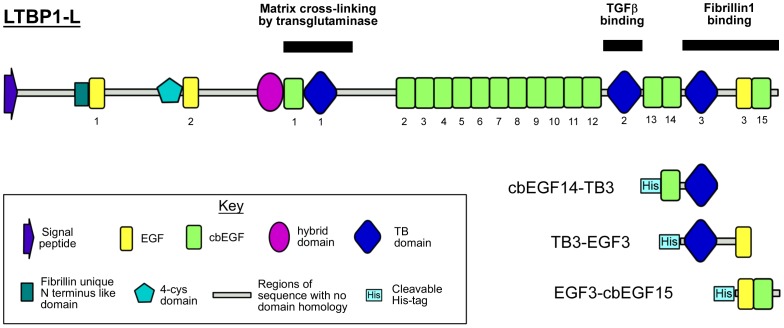
LTBP1 constructs. Three overlapping constructs, cbEGF14-TB3 (LTBP1L residues 1468–1582), TB3-EGF3 (LTBP1L residues 1509–1662) and EGF3-cbEGF15 (LTBP1L residues 1617–1722), were generated which span the fibrillin-1 binding region. The numbering of selected domains is also shown for clarity. Fragments were expressed prokaryotically and refolded *in vitro*. Some of the activities attributed to different regions of the LTBP1 molecule are highlighted by black lines.

The human genome encodes four latent transforming growth factor-β (TGFβ) binding proteins (LTBPs). These are large extracellular proteins which contain variable numbers of epidermal growth factor-like (EGF), calcium-binding EGF (cbEGF) and “TGFβ” binding protein-like (TB) domains, but all of which conform to a similar domain architecture (Figure 1). Significant regions of sequence with no known domain homologues are also present, especially at the N-terminus and C-terminus of the protein [1].

LTBPs play an important role in the TGFβ signalling pathway, which is a key component of many developmental and disease processes [2], [3]. Specifically LTBPs are involved in facilitating the folding and secretion of TGFβ from the cell [4], directing its deposition to the extracellular matrix (ECM) [5], [6], and also playing a role in its activation and release from the latent state [7], [8]. LTBP1 forms covalent disulphide links via its second TB domain to the propeptides of TGFβ 1, 2 and 3 (otherwise known as Latency Associated Propeptides (LAPs)) [9], [10].

LTBPs have been demonstrated to interact with components of the extracellular matrix including human fibrillin1 [11]–[14]. The minimum C-terminal fragment of LTBP1 that binds fibrillin1 in these studies starts at the third TB domain and continues to the C-terminus [11]–[14]. This interaction allows LTBP1 to sequester TGFβ to fibrillin microfibrils; the latter also bind other growth factors including bone morphogenic proteins (BMPs). Several lines of evidence point towards a physiologically important connection between the fibrillin matrix and TGFβ signalling. Marfan syndrome (MFS) is a genetic disease caused by autosomal dominant mutations in the fibrillin1 gene, which leads to developmental defects including tall stature, arachnodactyly, ectopia lentis, and aortic root enlargement and dissection [15]–[17]. Interestingly in both mouse models and human patients fibrillin haploinsufficiency has been demonstrated to lead to over activation of TGFβ [18], [19], and antagonism of TGFβ signalling in mouse models can restore normal development in many tissues [20]–[23]. However, the molecular and cellular mechanisms underlying this effect have yet to be fully elucidated.

The bioinformatic analysis and NMR experiments reported here were designed to investigate the domain organisation of the fibrillin1-binding region of LTBP1 which is adjacent to the LAP-binding TB2 domain. This is the first detailed study of the domain architecture of the LTBPs and reveals a dynamic “knotted rope” model for the C-terminus of LTBP1. This may have significant implications for its interaction with fibrillin and other ECM proteins, and also help decipher the role of LTBP1 in TGFβ regulation.

## Materials and Methods

### Construct cloning and protein production

Prokaryotic expression constructs were generated by cloning DNA fragments into the pQE30 expression vector (Qiagen), incorporating a His_6_ tag at the N-terminus. The EGF3-cbEGF15 protein was produced as described previously, spanning LTBP1L residues 1617–1722 [24]. LTBP1 cbEGF14-TB3 and TB3-EGF3 fragments were produced using a similar protocol (spanning LTBP1L residues 1468–1582, and 1509–1662 respectively), with the exception that calcium was not included in the refolding mixture for TB3-EGF3 and the His_6_ tag was not removed from TB3-EGF3 as this construct contained additional factor Xa sensitive sites. TB3-EGF3 was purified by anion exchange FPLC with the His_6_ tag still present. After purification all proteins were characterised by SDS-PAGE and Coomassie staining (Figure S1A), and the mass of purified proteins was confirmed by Electrospray Ionisation Mass spectrometry (data not shown).

### Nuclear magnetic resonance spectroscopy experiments

Experiments were carried out using home-built instruments operating at ^1^H frequencies ranging from 500 to 950 MHz. All experiments were carried out at 25°C. Data was processed using NMRpipe [25]. Spectra were analysed and figures generated using the CCPN Analysis software [26].

The spectra of cbEGF14-TB3 and TB3-EGF3 were assigned with ^15^N labelled protein, using 3D ^15^N-edited TOCSY experiments to identify spin systems, and 3D ^15^N-edited NOESY experiments to identify the through-space connections between residues. 3D experiments for resonance assignment of cbEGF14-TB3 were collected at 500 MHz while spectra for TB3-EGF3 were collected at 600 MHz. Backbone ^15^N and ^1^H^N^ assignments for cbEGF14-TB3 and TB3-EGF3 have been deposited in the BMRB with accession number 19322. The ^15^N-edited 3D data for EGF3-cbEGF15 were not of sufficient quality to allow complete backbone assignment. Therefore, assignment of the NMR spectrum of EGF3-cbEGF15 was carried out using ^13^C and ^15^N double-labelled protein as described previously [24]; these assignments have been deposited in the BMRB with accession number 18848.

Unless otherwise stated, all NMR experiments were carried out at 25°C in a 95% H_2_O: 5% D_2_O mixture. EGF3-cbEGF15 experiments were carried out at pH 5.4 with 10 mM calcium chloride and a protein concentration of 5 mM. Experiments on cbEGF14-TB3 were carried out at pH 5.9, with 20 mM calcium chloride and a protein concentration of 3 mM. TB3-EGF3 experiments were carried out at pH 6.9 with no calcium chloride or other salt present, and a protein concentration of 4 mM. Spectra for chemical shift comparisons were all collected at pH 6 with 10 mM calcium chloride.

Residual dipolar coupling (RDC) values for EGF3-cbEGF15 were obtained from IPAP spectra [27] collected in isotropic medium (95% H_2_O with 5% D_2_O, 5 mM calcium, at pH 5.4), and in a 3.5% PEG-hexanol liquid crystal alignment medium [28] (90% H_2_O, 10% D_2_O, 5 mM calcium at pH 5.4). RDC data were used to define the interdomain orientation of EGF3 and cbEGF15 as follows. The X-ray coordinates of human Notch-1 EGF12-EGF13 (PDB 2VJ3) [38] were used in the fitting procedure instead of the homology model. RDC values are extremely sensitive to the orientation of the H^N^-^15^N bond relative to the principle axes of alignment tensor and the energy minimisation carried out in the homology modelling procedure was found to alter atomic positions leading to poor fits. Relative domain orientation was determined as follows. First, the RDC values for the individual domains were fitted to the X-ray coordinates of individual domains, using an in-house program, to identify a subset of residues that gave a good fit. The overall fit between experimental and calculated RDC values was assessed using the Q value, defined as Q  =  [∑_i = 1,…,N_ (RDC^expt^ – RDC^calc^)^2^/N]^½^/RDC_rms_ [48]. Residues with poor fits (where the experimental and calculated RDC value differed by more than ∼7 Hz) generally were located in regions of the domain where the number of residues in loops between cysteines differed between LTBP1 EGF3 or cbEGF15 and the Notch structure; these were excluded from further fits. In the second phase, the subset of peaks from the two domains was fitted simultaneously to the X-ray structures of the Notch EGF12 and EGF13 domains but the relative orientation of the domains was allowed to change to optimise the fit (by minimising the Q value). Interdomain tilt angles were calculated using the program mod22 using the positions of residues within the major β-hairpin as the reference point [29] and an experimental RDC error of +/− 1 Hz to assess the error in the tilt angle.

Hydrogen/deuterium exchange experiments were used to provide information about amide solvent accessibility and hydrogen bonding in various constructs. In these experiments ^15^N-labelled protein samples were prepared at pH 5.9 with 20 mM calcium chloride, for cbEGF14-TB3, and at pH 5.4 with 10 mM calcium chloride, for EGF3-cbEGF15, and then freeze dried. These samples were then re-dissolved in 100% D_2_O (to the same volume as the sample prior to freeze drying), and a series of HSQCs collected over 24 hours to examine the loss of peak intensity.

^1^H-^15^N Heteronuclear NOE experiments were carried out on ^15^N-labelled protein samples in order to examine the sub-nanosecond dynamics of specific amides. Spectra with and without ^1^H saturation were collected as interleaved experiments. Error bars were calculated by taking the standard deviation of random background noise and recalculating NOE ratios after adding this standard deviation to the NOE peak intensities and subtracting it from the blank peak intensities.

### Homology modelling

Homology modelling was carried out using Modeller 9 v7 [30]. Modelling of LTBP1 cbEGF14-TB3 was based on the structure of fibrillin cbEGF22-TB4 [31] (PDB 1UZJ), but as there was no evidence found for an interface between LTBP1 cbEGF14 and TB3 domains (discussed in Results section), for illustrative purposes the two domains in this model were moved apart using the manual sculpting function in PyMOL and then re-modelled based on this template. The EGF3-cbEGF15 domain pair was modelled based on the fibrillin-1 cbEGF12-cbEGF13 domain pair [32] (PDB 1LMJ) (but with calcium excluded from LTBP1 EGF3). Both of these models were then combined and used as a Modeller template to generate an overall model of the LTBP1 C-terminus containing cbEGF14-TB3-EGF3-cbEGF15.

The LTBP1 TB2-cbEGF13 domain pair was modelled using the TB2 solution structure [33] (PBD 1KSQ) and a homology model of cbEGF13 based on the fibrillin 1 cbEGF23 domain [31], [34] (PDB 1UZJ). The relative orientation of TB2 and cbEGF13 was based on the orientation of the fibrillin1 TB4 and cbEGF23 domains (PDB 1UZJ); this is justified by the presence of similar packing residues in the TB and cbEGF domains. The relative orientation of LTBP1 cbEGF13 and cbEGF14 was based on the orientaton of fibrillin1 cbEGF32 and cbEGF33 [29].

### Bioinformatic techniques

Alignments were generated using the ClustaW feature of the MEGA5 suite of programs [35] with a BLOSUM matrix, a gap opening penalty of 15, and a gap extension penalty of 1. Alignments were then manually edited to ensure proper alignment of key sequence features. For display purposes alignments were imported into the PFAAT alignment viewer [36] and coloured accordingly.

## Results

### Alignments show clear conservation of C-terminal domains, but poor conservation of linker regions and inter-domain packing residues

**Figure 2:**
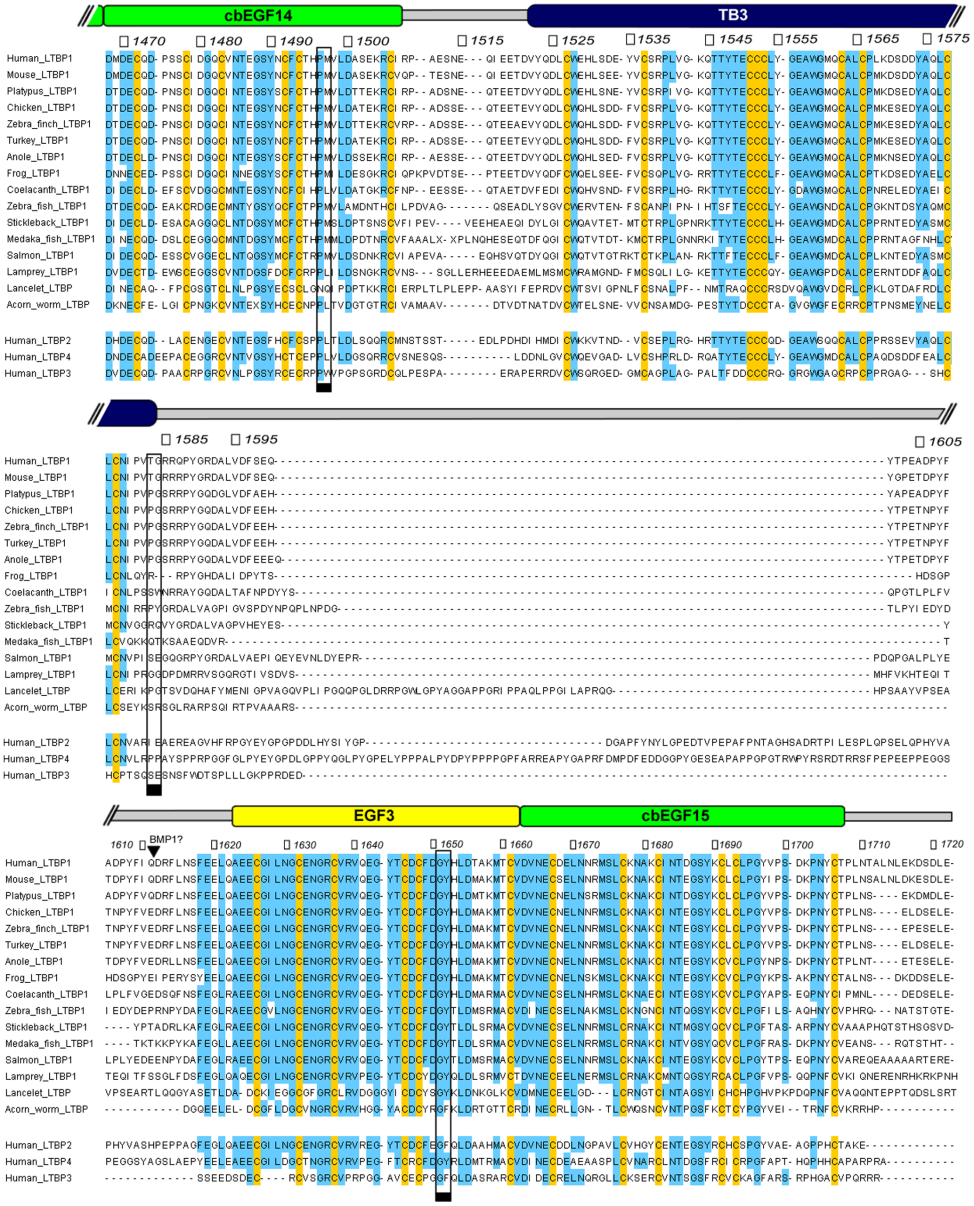
Alignment of cbEGF14-TB3-EGF3-cbEGF15 domains of LTBP1 and LTBP-like proteins from a variety of organisms. Cysteine residues coloured yellow are found in 100% of the aligned sequences. Residues coloured blue are found in 70% or more of the aligned sequences. Numbers at the top of the alignment refer to residue numbers of human LTBP1. Black boxes highlight sites where interdomain packing G(Y/F) motifs would be expected in cbEGF14, TB3, and EGF3 domains (for packing with the C-terminal domain). A black arrow labelled “BMP1?” highlights a potential BMP1 cleavage site.

LTBP and true TGFβ first emerged with the evolution of deuterostomes [1], [7]. C-terminal LTBP sequences from a diverse range of these organisms were collected and aligned to assess the conservation of specific residues and domains (Figure 2). TB/EGF/cbEGF domains were identified by their expected distribution of cysteine residues and additional conserved residues associated with each fold. The cbEGF-TB-EGF-cbEGF C-terminal domain arrangement of LTBP1 appears conserved in all other complete LTBP-like protein sequences found, including the acorn worm, which represents one of the most evolutionarily distant deuterostomes where LTBP and true TGFβ are observed. The EGF3 equivalent domains consistently lack calcium binding consensus sequences, while domains equivalent to cbEGF14 and cbEGF15 consistently have these residues, suggesting a biologically conserved role for calcium binding at these positions in the C-terminal region.

LTBP sequences all contain linker regions of 17 or more amino acids between the cbEGF14 and TB3 domains, and 34 or many more residues between the TB3 and EGF3 domains. These long linker regions have no clear homology to previously identified domains and are not seen between homologous TB/EGF/cbEGF domains in fibrillin.These sequences are poorly conserved between LTBP variants, suggesting that these regions may be unstructured and potentially flexible. Interdomain packing interactions between adjacent TB and cbEGF domains have been described previously and are often centred around specific glycine-aromatic motifs in the N-terminal (cb)EGF or TB domain that form non-polar interactions with the C-terminal (cb)EGF domain [34]. There is a notable lack of glycine-aromatic interdomain packing motifs in both the cbEGF14 and TB3 domains (normal motif positions are boxed in Figure 2). The glycine-aromatic motifs in EGF3, on the other hand, are highly conserved, suggesting a packing interaction may occur between EGF3 and cbEGF15.

Homology models were generated for the four domains of LTBP1 cbEGF14-TB3-EGF3-cbEGF15 by assuming that these domains adopt the canonical domain folds seen for other (cb)EGF and TB domain structures [29], [31]-[33], [37]; which was justified on the basis of bioinformatic analysis. The validity of these models was then assessed using a variety of NMR data.

### Resonance assignment and confirmation of domain folds

To test the hypotheses of LTBP1 domain structure and linker flexibility suggested by bioinformatics analysis, three overlapping protein fragments spanning from the N-terminus of cbEGF14 to the C-terminus of LTBP1 were produced (Figure 1). These fragments contain domain pairs cbEGF14-TB3, TB3-EGF3 and EGF3-cbEGF15.

To facilitate chemical shift assignment proteins were isotopically labelled; ^15^N labelling alone was used for the cbEGF14-TB3 and TB3-EGF3 assignments, while EGF3-cbEGF15 was assigned with ^13^C and ^15^N labelling as described previously [24] (BMRB accession 18848). Xa-cleaved cbEGF14-TB3 contains 117 amino acids; 88% of the backbone amides were assigned using 3D ^15^N-edited NOESY and TOCSY experiments (BMRB accession 19322). TB3-EGF3 was the largest fragment assigned with 172 amino acids (including a His_6_ tag); 78% of the backbone amides were assigned (BMRB accession 19322). Comparison of HSQC spectra from the three LTBP1 fragments shows that residues present in more than one construct give rise to peaks with very similar chemical shifts in the relevant HSQC spectra (Figure S1B). This confirms that the TB3 domain adopts the same fold in cbEGF14-TB3 and TB3-EGF3, and that the EGF3 domain adopts the same fold in TB3-EGF3 and EGF3-cbEGF15.

**Figure 3:**
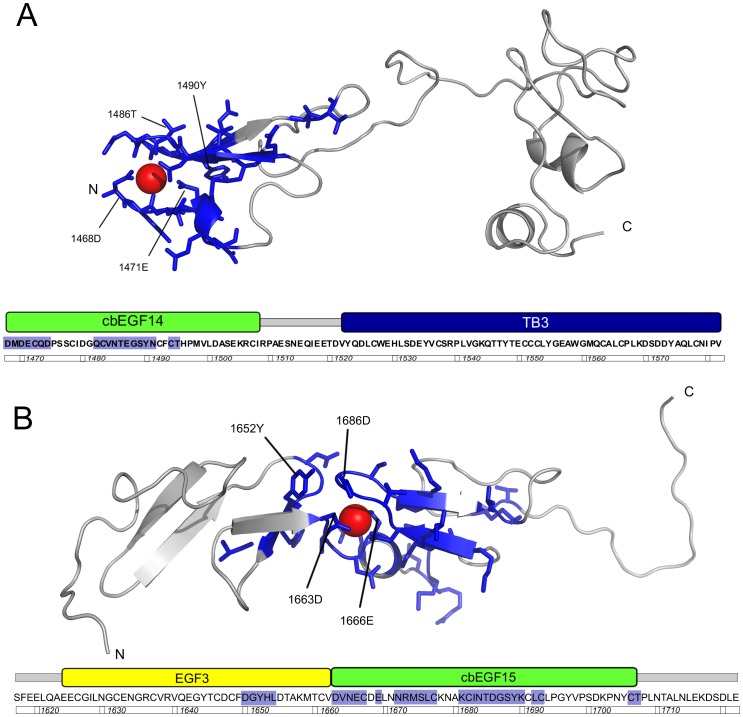
Residues in cbEGF14-TB3 and EGF3-cbEGF15 affected by calcium binding. Calcium-dependant chemical shifts mapped onto homology models of A) cbEGF14-TB3 and B) EGF3-cbEGF15. Residues with calcium-dependant chemical shifts are shown in stick representation and coloured blue. Calcium is also shown as a red sphere in both structures.

The binding of Ca^2+^ to a cbEGF domain is accompanied by significant chemical shift changes [38]. To assess calcium binding in cbEGF14 and cbEGF15, HSQC spectra of cbEGF14-TB3 and EGF3-cbEGF15 were collected in the presence of different calcium concentrations (Figure S2). For the cbEGF14-TB3 fragment, a number of peaks exhibited significant shifts in response to increasing calcium concentration, and the corresponding residues are highlighted in the homology model (Figure 3A). The peaks exhibiting the largest shifts correspond to residues at the N-terminus of cbEGF14 and in the main β-hairpin, suggesting that the homology model provides an accurate representation of the calcium-binding site. In this titration cbEGF14 did not reach saturation upon addition of 20 mM calcium, demonstrating a low calcium affinity (K_d_>1 mM) typical of cbEGF domains in an N-terminal position. In native LTBP1, cbEGF13 would be expected to pack against cbEGF14 leading to an increased calcium affinity in cbEGF14.

In EGF3-cbEGF15, by contrast, the N-terminus of cbEGF15 is in a more native context, preceded by EGF3. A calcium titration of this construct monitored by 1D NMR shows slow exchange behaviour characteristic of high-affinity calcium binding (K_d_<∼25 µM); some peaks, corresponding to the calcium-bound form, appear and strengthen as calcium is added, while others, corresponding to the calcium-free state, weaken and disappear (Figure S2B inlay). HSQC spectra collected in the presence and absence of calcium (Figure S2B) allow identification of calcium-sensitive peaks; these correspond to residues near the calcium-binding site of cbEGF15 and a stretch of residues at the C-terminus of EGF3 (Figure 3B). These EGF3 residues comprise the loop containing the “GY” interdomain packing motif, providing evidence for a packing interaction between this region of EGF3 and the calcium-binding region of cbEGF15.

The homology models of all four domains contain hydrogen-bonded secondary structure. The involvement of specific amides in the predicted hydrogen bonds was probed using hydrogen/deuterium (H/D) exchange monitored by HSQC spectra following dissolution of the protein fragments in D_2_O. The cbEGF14-TB3 construct demonstrated a number of slowly exchanging amide protons in D_2_O (Figure S3A). Three of these correspond to residues in the cbEGF14 domain predicted to be hydrogen bonded in the main β-hairpin and C-terminal loop of the domain. The remaining slowly-exchanging amides correspond to residues in the TB3 domain with predicted hydrogen bonds in α-helical and anti-parallel β-sheet regions. The most slowly exchanging amides correspond to residues located within the hydrophobic core of the TB3 domain. EGF3-cbEGF15 also demonstrated a number of slowly-exchanging amides (Figure S3B) located in both EGF3 and cbEGF15; these correspond well with the pattern of hydrogen bonding predicted by the homology model, and many of the most protected amides are found in the main β-hairpins of both EGF3 and cbEGF15. The H/D exchange data strongly support the homology models for the four domains.

### Fast timescale dynamics demonstrate flexible inter-domain linkers between the cbEGF14 and TB3 domains and between the TB3 and EGF3 domains

**Figure 4:**
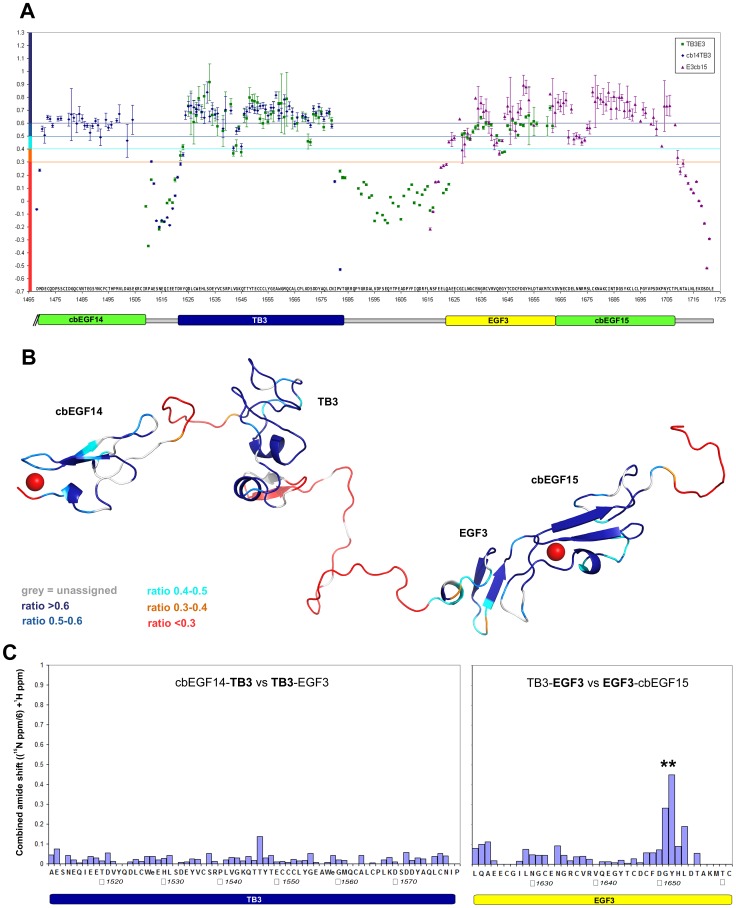
Dynamics and packing interactions of the LTBP1 C-terminus, as determined by heteronuclear NOE experiments and comparison of HSQC peak positions in overlapping constructs. A) Heteronuclear NOE ratios observed for residues in the cbEGF14-TB3 (dark blue), TB3-EGF3 (green) and EGF3-cbEGF15 (purple) constructs. A summary of the domain organisation is shown below. Error bars give the standard error for each value based on peak intensity relative to background noise. The heteronuclear NOE values for EGF3 are higher in the EGF3-cbEGF15 construct than in the TB3-EGF3 construct. In EGF3-cbEGF15, the presence of a well-defined packing interface means that the pair of domains tumbles as a single unit. In TB3-EGF3, where no interface is observed for the two domains, EGF3 tumbles as a smaller independent domain. B) Heteronuclear NOE values from A) plotted on a homology model of the complete LTBP1 C-terminus, with linker regions placed randomly using the PyMOL manual sculpting tool. Colour code provided at the bottom left, for overlapping residues 1488–1578 values from cbEGF14-TB3 are used, for residues 1579–1648 values from TB3-EGF3 are used, and for residues 1649–1722 values from EGF3-cbEGF15 pair are used. C) Differences in HSQC peak positions between pairs of constructs are plotted for the TB3 and EGF3 domains and inform on the presence or absence of inter-domain packing sites. Generally, only small differences in peak position are seen, the exception is in the GY packing motif of EGF3 (highlighted with **). This demonstrates the absence of inter-domain packing sites in TB3 and supports a packing interaction between EGF3 and cbEGF15.

To determine whether stretches of amino acids connecting cbEGF14 to TB3 and TB3 to EGF3 adopt rigid structures or instead act as flexible interdomain linkers, ^1^H-^15^N heteronuclear NOE experiments, which report on fast-timescale backbone dynamics, were carried out on all three protein fragments (Figure 4A). Different patterns of heteronuclear NOE ratios are observed within the well-defined domains and the linker regions. Heteronuclear NOE ratios of ∼0.6-0.8, consistent with a relatively rigid backbone structure, are observed for residues corresponding to the cbEGF14, TB3, EGF3 and cbEGF15 domains. For residues in the linkers connecting cbEGF14 to TB3 and TB3 to EGF3 and for the 13 C-terminal residues following cbEGF15, NOE ratios below 0.3 are observed, indicating significant amplitude backbone dynamics on a sub-nanosecond timescale. This high degree of flexibility and lack of well-defined structure is consistent with the poor sequence conservation of these regions (Figure 2).

As well as the large unstructured regions in the LTBP1 C-terminus there are a number of flexible loops within individual domains. Residues in cbEGF14 appear well structured, except for the two N-terminal residues; these may be stabilised in a native context when preceded by cbEGF13. The structured region of the TB3 domain starts from Gln 1524 and finishes with Asn 1579, which follows immediately after the last cysteine of the domain. While most of the TB3 backbone appears structured a small stretch of amino acids from Val 1542 to Gln 1545, forms a flexible loop, predicted to be at the surface of the TB domain (Figure 4B highlighted in lighter blue). Overall the EGF3 domain is structured, but does exhibit some regions of increased flexibility (heteronuclear noe ratio <0.5), most notably in the turn in the main β-hairpin (residues 1640-1644). In contrast, the turn in the main β-hairpin of cbEGF15 gives consistently high heteronuclear NOE values, presumably due to the stabilising effect of Ca^2+^ binding, and only the loop region between the first and second cysteines exhibits significant flexibility in this domain. The five residues in between the last cysteine of EGF3 and the first cysteine of cbEGF15 give high heteronuclear NOE ratios indicating that they are not flexible on a fast time scale; this is consistent with a packing interaction between these two domains.

### Comparison of chemical shifts to identify inter-domain interfaces in the LTBP1 C-terminus

The presence of flexible interdomain linkers between cbEGF14 and TB3, and between TB3 and EGF3, suggests a model of a flexible LTBP1 C-terminus, with independent movement of the TB3 domain and EGF3-cbEGF15 domain pair relative to each other and the rest of the LTBP1 molecule. However the presence of these flexible linkers by themselves is not conclusive evidence for this model, as interdomain interactions could still occur, with the intermediate linker regions acting as extremely large flexible loops protruding from interacting domains.

However, comparison of the NMR spectra of the constructs shows very little change in the chemical shift of peaks from domains present in overlapping constructs (Figure 4C). The exception to this are the peaks from the glycine-aromatic packing motif of EGF3 (* in Figure 4C). The chemical shift analysis demonstrates the absence of inter-domain packing sites in TB3, and supports a packing interaction between EGF3 and cbEGF15. The lack of other interfaces supports a model of a dynamic and flexible LTBP1 C-terminus, with independent movement of the TB3 domain and EGF3-cbEGF15 domain pair.

### RDC measurements support an elongated rod-like structure for EGF3-cbEGF15

The results from the calcium titration, heteronuclear NOE experiments and chemical shift analysis all support interface formation between the LTBP1 EGF3 and cbEGF15 domains. However, they do not provide direct information about the relative orientations adopted by the two domains as a result of this interface. Structures of cbEGF-cbEGF pairs from fibrillin [29], [32] and Notch [39] show a linear, elongated arrangement of the domains, but with some variation in interdomain angles. The EGF2-EGF3 pair of fibrillin 1 also demonstrates a linear arrangement [40], however other EGF-EGF pairs have non-linear arrangements. For example, the first and second EGF domains of Developmental endothelial cell locus-1 (Del-1) glycoprotein display a near 90° bend at the EGF-EGF interface [41], and the third and fourth EGF domains of vitamin K-dependent protein S have been shown to interchange between elongated and bent conformations [42].

**Figure 5:**
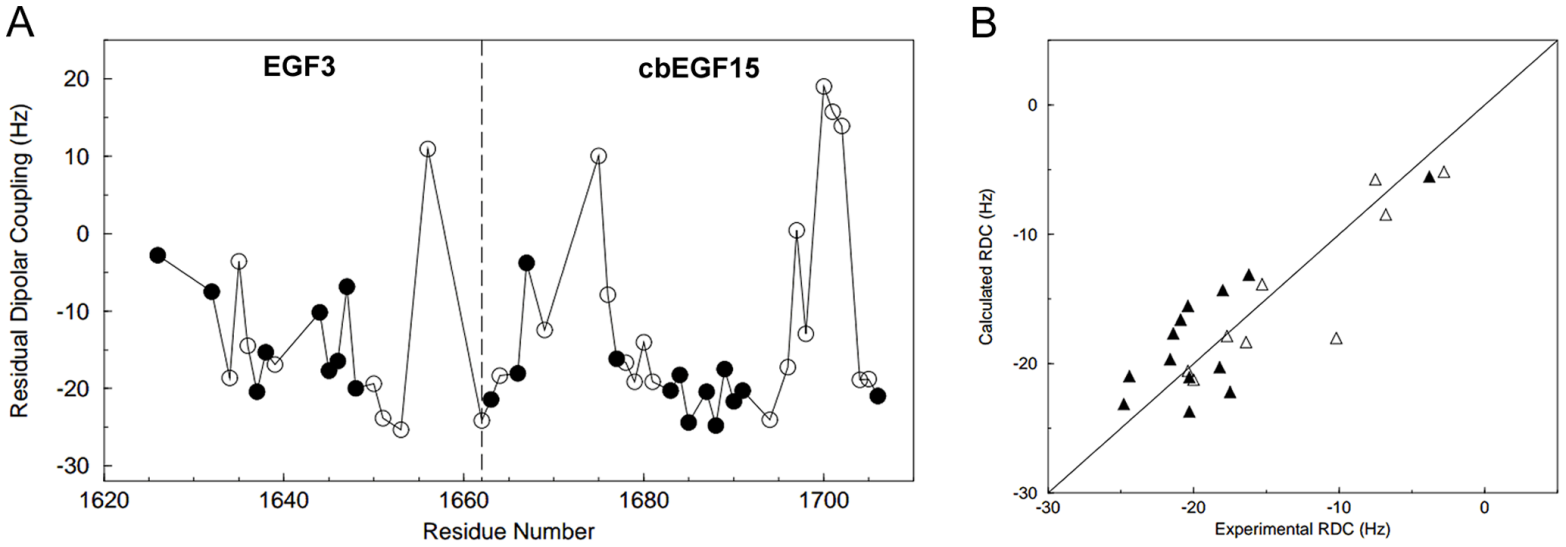
RDC analysis of the LTBP1 EGF3-cbEGF15 domain pair. A) Experimentally measured RDC values for EGF3-cbEGF15 are plotted as a function of sequence. Filled circles indicate the RDC values that were used in the fitting procedure to determine the inter-domain orientation of EGF3 and cbEGF15. The dotted line shows the boundary between EGF3 and cbEGF15. B) Plot of experimental RDCs versus RDCs obtained from the fitting procedure. Residues from EGF3 are shown as open triangles and those from cbEGF15 as filled triangles. The good correlation between experimental and calculated RDC values (R = 0.86) indicates that the RDC data are consistent with a rigid structure for EGF3-cbEGF15 with a well-defined inter-domain orientation.

The relative orientation of EGF3 and cbEGF15 in LTBP1 were investigated using Residual Dipolar Couplings (RDCs). RDCs were measured for 18 and 30 residues in EGF3 and cbEGF15, respectively (Figure 5A). The large RDCs measured for this domain pair, ranging from +20 to −25 Hz, in 3.5% PEG/hexanol is suggestive of a relatively elongated structure. The X-ray coordinates of EGF12-EGF13 from human Notch-1 were used to fit the RDC data. The EGF12-EGF13 structure was selected as a model because these domains have the same number of residues in the major β-hairpin as EGF3 and cbEGF15. A subset of 9 and 13 RDC values from EGF3 and cbEGF15, respectively, were used in the fits as described in Materials and methods. RDCs for EGF3 were predicted well using the EGF12 structure; a Q value of 0.09 was obtained. Similarly, RDCs for cbEGF15 were successfully predicted using the EGF13 structures; a Q value of 0.13 was obtained. When the two sets of RDCs are fitted simultaneously using the X-ray coordinates and the inter-domain orientation is optimised during the fitting procedure, a Q value of 0.18 is obtained (Figure 5B). In this fitted structure the interdomain tilt angle is found to be 26±5° indicating a nearly linear arrangement of the domains. In fibrillin-1 cbEGF-cbEGF structures determined previously by NMR the tilt angle is found to be 18±6° for cbEGF32-33 [29] and 30±15° for cbEGF12-13 [32].

## Discussion

These NMR studies have validated structural models for the four C-terminal domains of LTBP1 and given significant insight into the overall dynamics and interdomain interactions of this region. The numerous flexible linkers identified would make many regions of the LTBP1 C-terminus unsuitable for crystallographic studies, or other structural investigations that rely on rigid body modelling. The data presented here demonstrate that the C-terminus of LTBP1 behaves like a “knotted rope” in solution, where the linkers either side of the TB3 domain act as the highly flexible “rope” allowing the TB3 domain and EGF3-cbEGF15 domain pair, (the “knots” in this analogy), to move freely relative to each other and the rest of LTBP1. The presence of flexible linkers mean that long-range interdomain interactions, such as EGF3 interacting with cbEGF14 or other parts of the LTBP1 molecule, cannot be excluded by these studies.

Bioinformatics observations support this model, and also demonstrate that the structural and dynamic properties of the LTBP C-terminus may have emerged prior to the divergence of acorn worms and the other deuterostomes over 500 million years ago [43]. The consistent presence of key sequence features suggests that this knotted rope-like structure is important for the biological function of LTBPs, as these features have not been lost over a significant evolutionary time-frame. At present it is not clear precisely how these sequence features and their structural properties relate to LTBP1 function. The only activity determined for the LTBP1 C-terminus so far is interaction with the extracellular matrix via fibrillin microfibrils [11]–[14]. The structural information provided here will be of significant use for developing a greater understanding of this interaction. The minimal fragment of LTBP1 that has been shown to bind the N-terminus of fibrillin1 spans from the TB3 domain to the C-terminus, and the structural model proposed here covers the entirety of this region (Figure 1).

**Figure 6:**
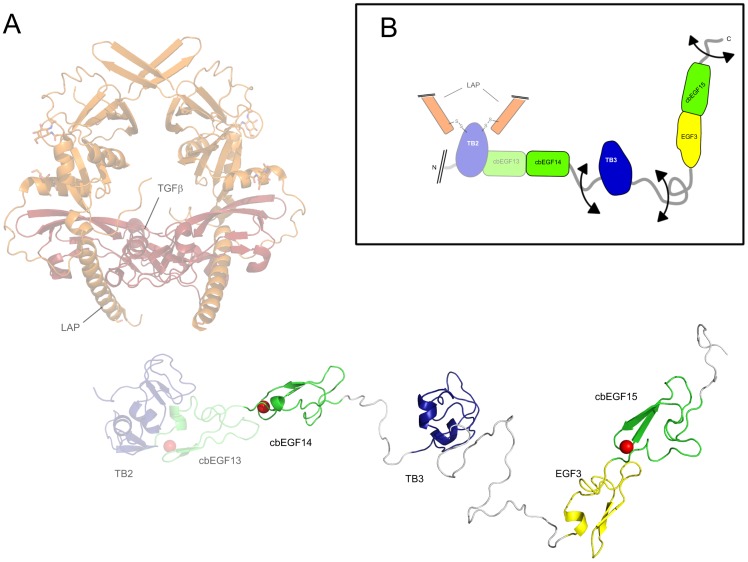
Current model for the structure of the LTBP1 C-terminus. A) Homology model of the LTBP1 C-terminus (coloured as in Figure 1A), and the structure of TGFβ1 (coloured dark red) in complex with its propeptide (coloured orange) [46], both shown on the same scale (rendered using PyMOL). For context the LTBP1 TB2 domain and cbEGF13 domain are also included, along with the four domains studied here, but rendered semi transparently. B) Cartoon representation of the LTBP1 C-terminus using arrows to highlight regions of significant flexibility.

Several proteins such as fibulin4 [12], and ADAMTSL proteins [44] have been proposed to form ternary complexes with LTBP1 and fibrillin, although their binding sites have not yet been defined. A reason for the conservation of flexible interdomain linkers through evolution may be that they facilitate multiple protein interactions by allowing rearrangements of LTBP1, in order to accommodate the potentially diverse orientations of other factors, possibly tissue specific, bound to the fibrillin microfibril. It may also be relevant to TGFβ activation, as before TGFβ can bind its receptors and induce signalling cascades, it must be released from the large latent complex with its propeptide and LTBP [45]. The crystal structure of TGFβ1 bound to its pro-peptide has been recently determined [46], and this is shown in Figure 6 along with a structural model of the LTBP1 C-terminus. From this figure it can be seen that the LTBP1 C-terminus would have more than sufficient flexibility to interact directly with the TGFβ and its pro-peptide, or allow interactions of TGFβ with other factors bound to this region. The presence of flexible interdomain linkers also explains the protease sensitivity of these regions that may play a role in TGFβ activation [47].

Interestingly LTBP2 and LTBP4 have even longer linkers joining their equivalents of LTBP1's TB3 and EGF3 domains (Figure 2). While rich in aromatic and proline residues the linkers in LTBP2 and LTBP4 do not contain any clear structural motifs, and it is conceivable that they are also flexible. These longer linkers are consistently seen in LTBP2 and LTBP4 like proteins from other species, and this may relate to unique functional properties of these LTBPs. The same argument can also be made for LTBP3 like proteins which have consistently shorter linkers than LTBP1, which may also endow LTBP3 with unique functional properties (Figure 2).

The work presented here establishes for the first time the structural organisation for the C-terminus of LTBP1, a molecule of significant importance in TGFβ regulation. These results will help guide further studies to understand the complex relationship between TGFβ signalling and ECM localisation, and reveal the true relevance of LTBP1's knotted tail.

## Supporting Information

Figure S1 **Protein characterisation.** A) Protein products analysed by SDS-PAGE and Coomassie staining, under both non-reducing and reducing conditions. The cbEGF14-TB3 fragment migrated unusually far under non-reducing conditions, especially when compared with reducing conditions, this behaviour was seen reproducibly, and may reflect unusual behaviour of this fragment in the presence of SDS and electric fields. Mass spectrometry confirmed the protein was of the expected size in each case. Bi-iii) 500 MHz HSQC spectra of all three constructs, collected at pH 6. Some example peaks corresponding to the TB3 domain are highlighted with blue circles in the cbEGF14-TB3, and TB3-EGF3 spectra, while some peaks corresponding to EGF3 residues are highlighted with orange circles in the TB3-EGF3 and EGF3-cbEGF15 spectra.
(TIF)

Figure S2 **Calcium sensitivity of the HSQC spectra of cbEGF14-TB3 and EGF3-cbEGF15.** A) Overlaid 500 MHz HSQC spectra of 2 mM cbEGF14-TB3 collected with different concentrations of calcium, coloured according to the legend in the top left. The sample was prepared at pH 5.9, in 95% H_2_O : 5% D_2_O without any additional salt. Assignments for peaks showing significant shifts are highlighted. B) 750 MHz HSQC spectra of 5 mM EGF3-cbEGF15 recorded with and without 10 mM calcium at pH 5.4. The inlay in the bottom right shows the upfield shifted methyl region of 1D spectra of a 2 mM EGF3-cbEGF15 sample, collected at different calcium concentrations. The appearance of new peaks rather than shifts demonstrates slow exchange between the calcium-free and calcium-bound states of the protein (1D spectra collected in D_2_O at pH 7.7 and 750 MHz).
(TIF)

Figure S3 **Slowly-exchanging amides mapped onto the homology models of cbEGF14-TB3 and EGF3-cbEGF15.** Slowly-exchanging amides in cbEGF14-TB3 (A) and EGF3-cbEGF15 (B) highlighted in the protein sequence, and as blue spheres on the homology model.
(TIF)
