# Supplementary figures and images for: NMR Spectroscopic and Bioinformatic Analyses of the LTBP1 C-Terminus Reveal a Highly Dynamic Domain Organisation
Source: PLoS One. 2014 Jan 29;9(1):e87125. doi: 10.1371/journal.pone.0087125 (PMC3906135; doi:10.1371/journal.pone.0087125)

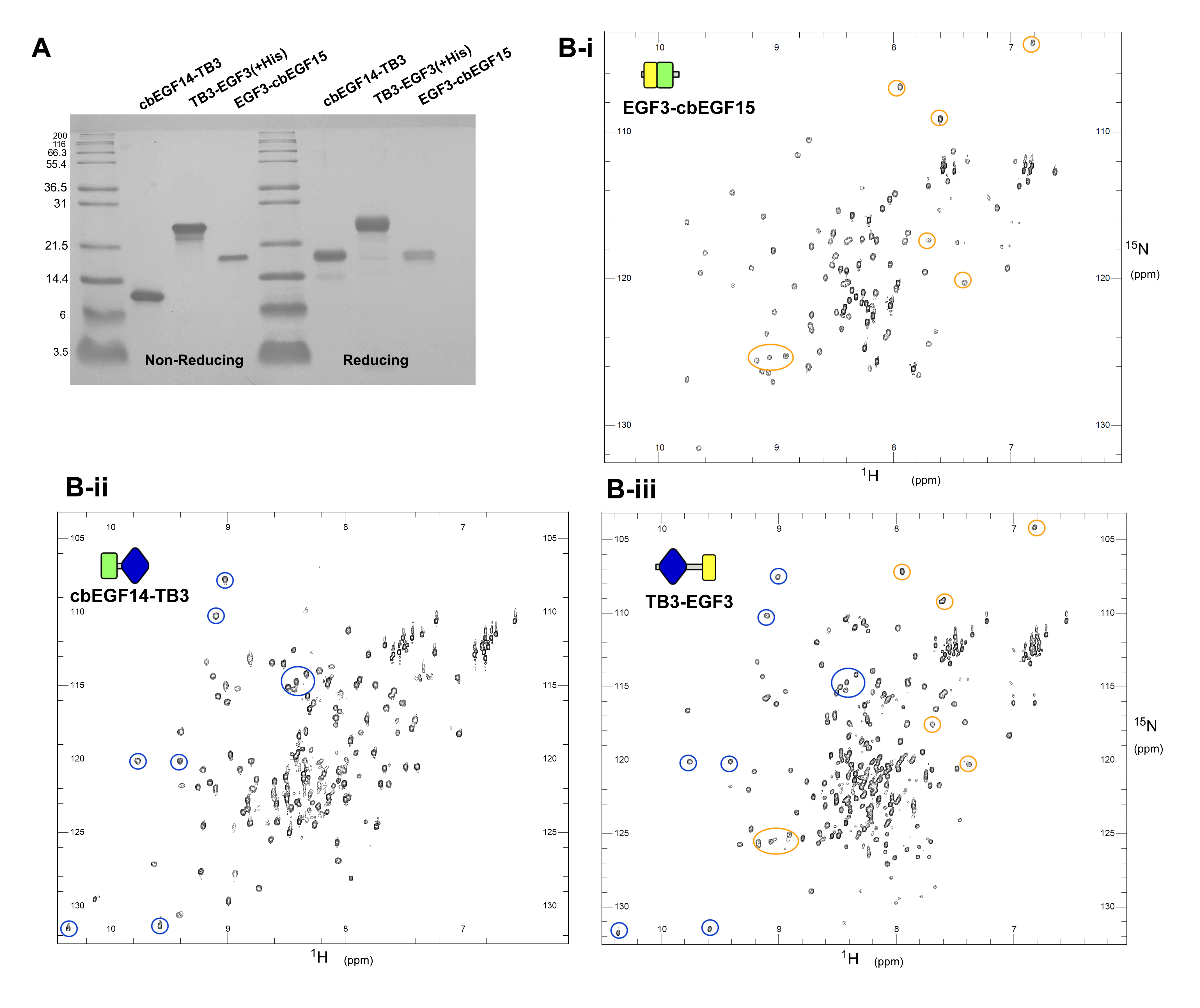

Supplement: Figure S1 — Protein characterisation. A) Protein products analysed by SDS-PAGE and Coomassie staining, under both non-reducing and reducing conditions. The cbEGF14-TB3 fragment migrated unusually far under non-reducing conditions, especially when compared with reducing conditions, this behaviour was seen reproducibly, and may reflect unusual behaviour of this fragment in the presence of SDS and electric fields. Mass spectrometry confirmed the protein was of the expected size in each case. Bi-iii) 500 MHz HSQC spectra of all three constructs, collected at pH 6. Some example peaks corresponding to the TB3 domain are highlighted with blue circles in the cbEGF14-TB3, and TB3-EGF3 spectra, while some peaks corresponding to EGF3 residues are highlighted with orange circles in the TB3-EGF3 and EGF3-cbEGF15 spectra. (TIF) [file pone.0087125.s001.tif]

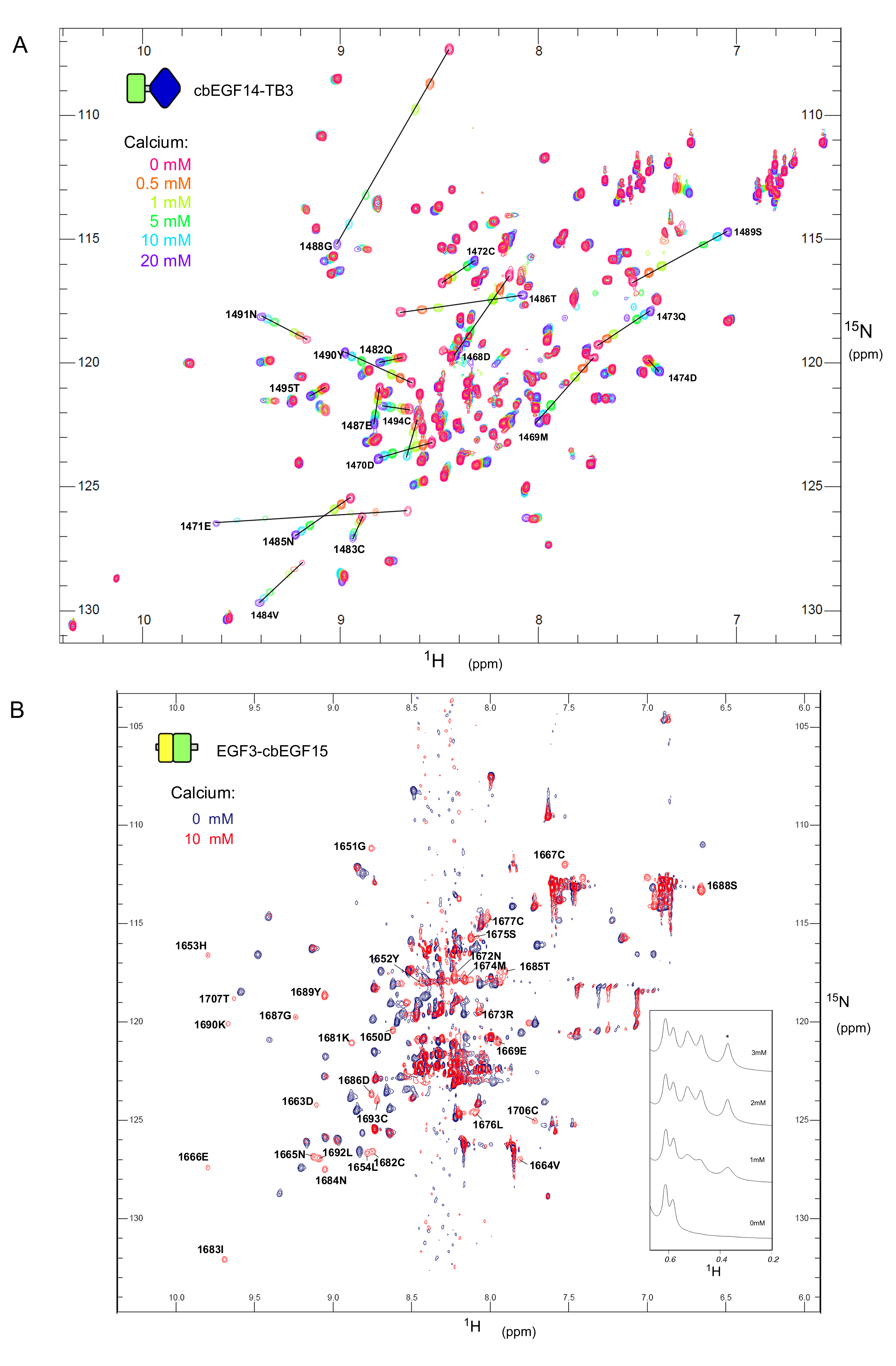

Supplement: Figure S2 — Calcium sensitivity of the HSQC spectra of cbEGF14-TB3 and EGF3-cbEGF15. A) Overlaid 500 MHz HSQC spectra of 2 mM cbEGF14-TB3 collected with different concentrations of calcium, coloured according to the legend in the top left. The sample was prepared at pH 5.9, in 95% H2O : 5% D2O without any additional salt. Assignments for peaks showing significant shifts are highlighted. B) 750 MHz HSQC spectra of 5 mM EGF3-cbEGF15 recorded with and without 10 mM calcium at pH 5.4. The inlay in the bottom right shows the upfield shifted methyl region of 1D spectra of a 2 mM EGF3-cbEGF15 sample, collected at different calcium concentrations. The appearance of new peaks rather than shifts demonstrates slow exchange between the calcium-free and calcium-bound states of the protein (1D spectra collected in D2O at pH 7.7 and 750 MHz). (TIF) [file pone.0087125.s002.tif]

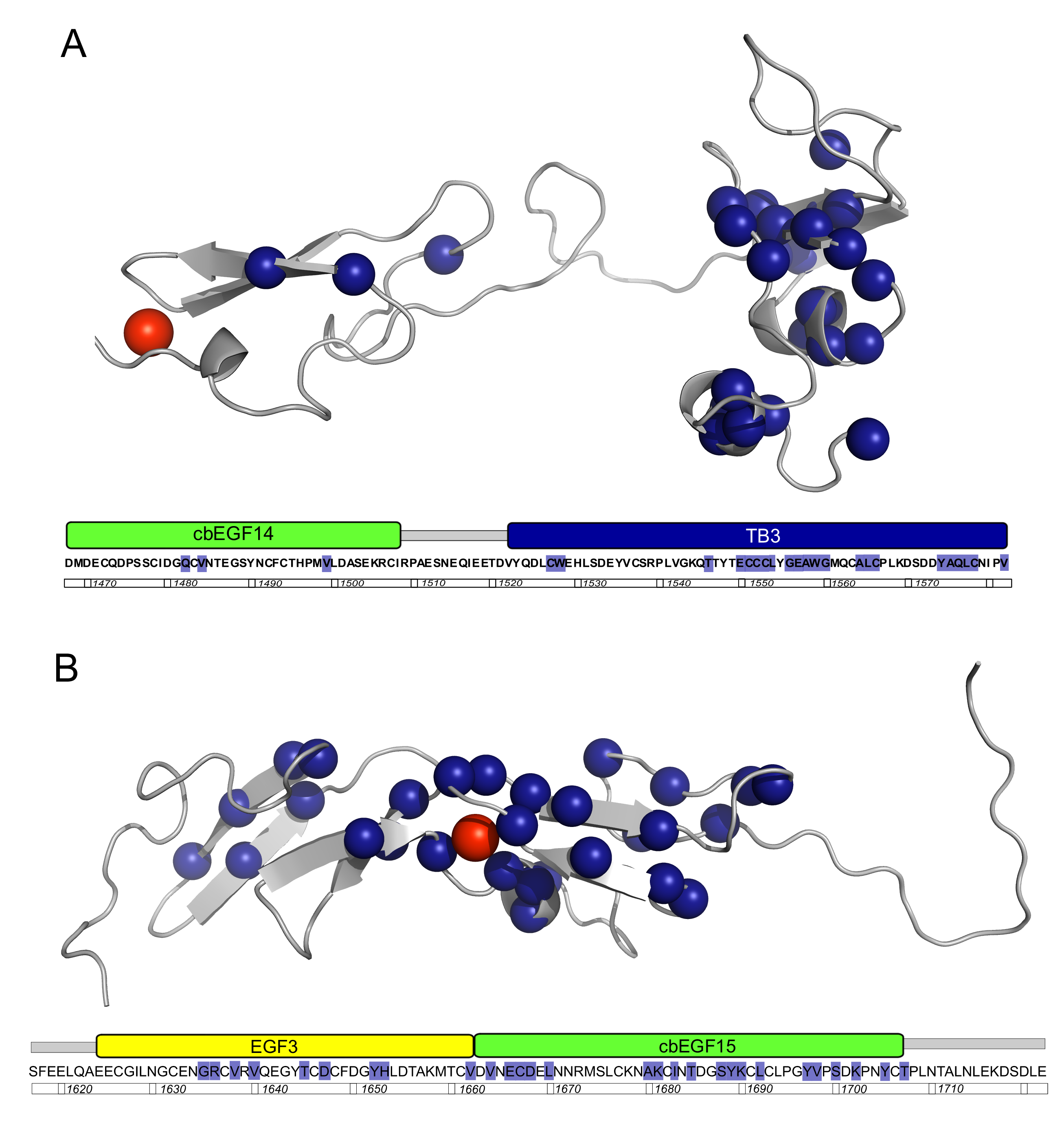

Supplement: Figure S3 — Slowly-exchanging amides mapped onto the homology models of cbEGF14-TB3 and EGF3-cbEGF15. Slowly-exchanging amides in cbEGF14-TB3 (A) and EGF3-cbEGF15 (B) highlighted in the protein sequence, and as blue spheres on the homology model. (TIF) [file pone.0087125.s003.tif]
